# Supplementary material for: Three-dimensional Pentagon Carbon with a genesis of emergent fermions
Source: Nat Commun. 2017 Jun 5;8:15641. doi: 10.1038/ncomms15641 (PMC5465352; doi:10.1038/ncomms15641)
Supplement: Supplementary Information — Supplementary Figures, Supplementary Notes and Supplementary References [file ncomms15641-s1.pdf]

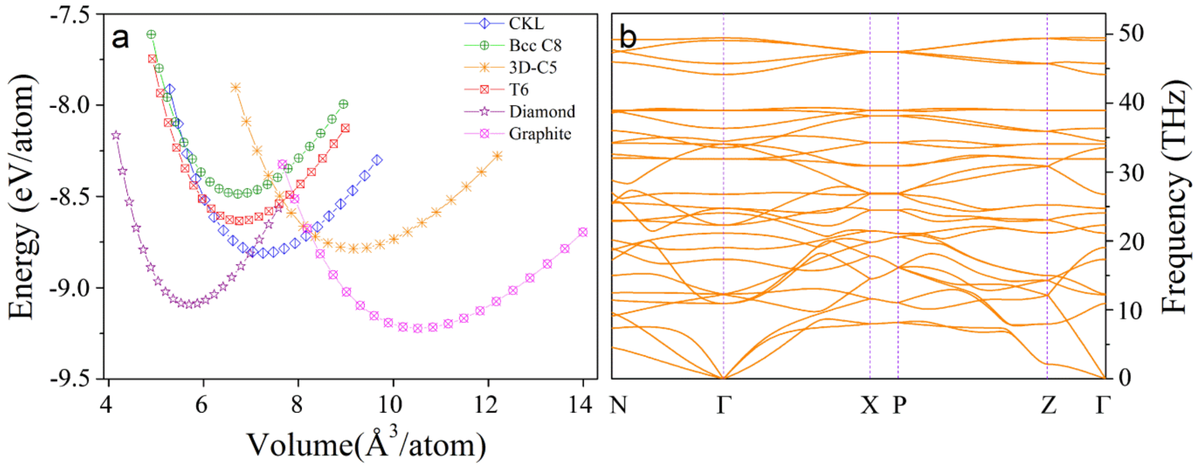

Supplementary Figure 1 | Energetic and dynamic stability of Pentagon Carbon. (a) Total energy per atom as a function of volume (equation of states, EOS) for carbon allotropes, including CKL, Bcc C8, 3D-C5, T6, Diamond, and Graphite. The EOS for each carbon structure has a local minimum, implying its state is energetic (meta-) stable. (b) Phonon dispersion of 3D-C5. There are no imaginary phonons vibrational frequencies, indicating that the structure is dynamically stable.

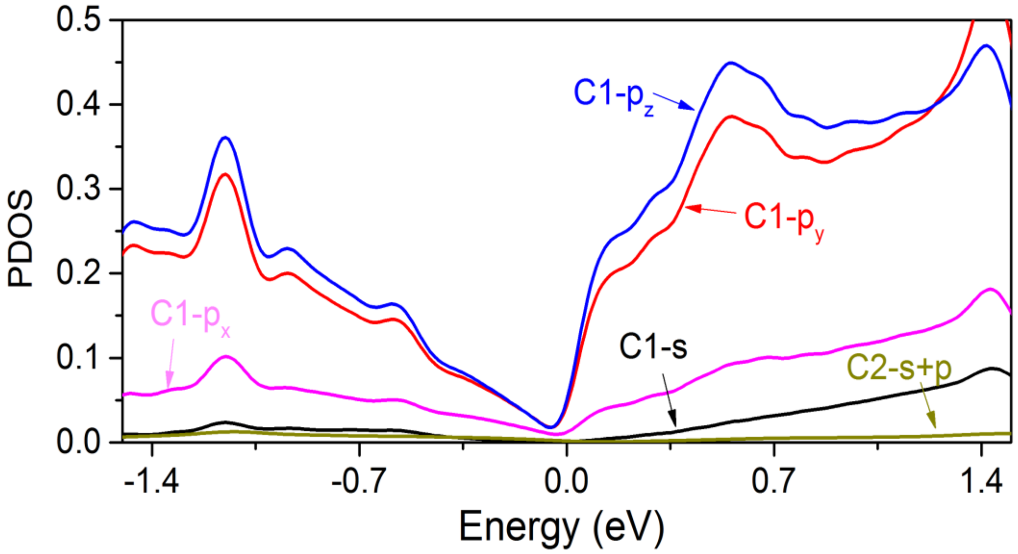

Supplementary Figure 2 | Orbital-resolved projected density of states (PDOS) near the Fermi level. The PDOS identifies the orbital contribution from each atom in the unit cell. The information is used for constructing the tight-binding model.

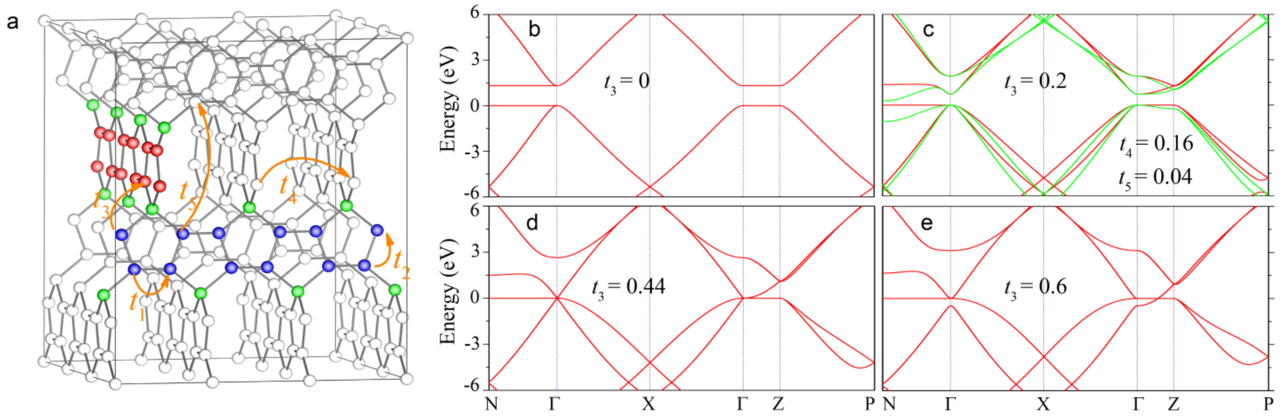

Supplementary Figure 3 | Tight-binding model and its band structures. (a) The TB model and the corresponding hopping energies. (b-e) The red lines are the results for t_3 = 0, 0.2, 0.44 and 0.6 eV, respectively. In all case, t_1 = 3.04 eV and t_2 = -2.6 eV. The green lines in (c) are the results of including t_4 and t_5. According to the analytical formula, when $|t_1+t_2| > (=, \text{or} <) |t_3|$, the energy difference Δ (defined in the main text) is positive (zero, or negative).

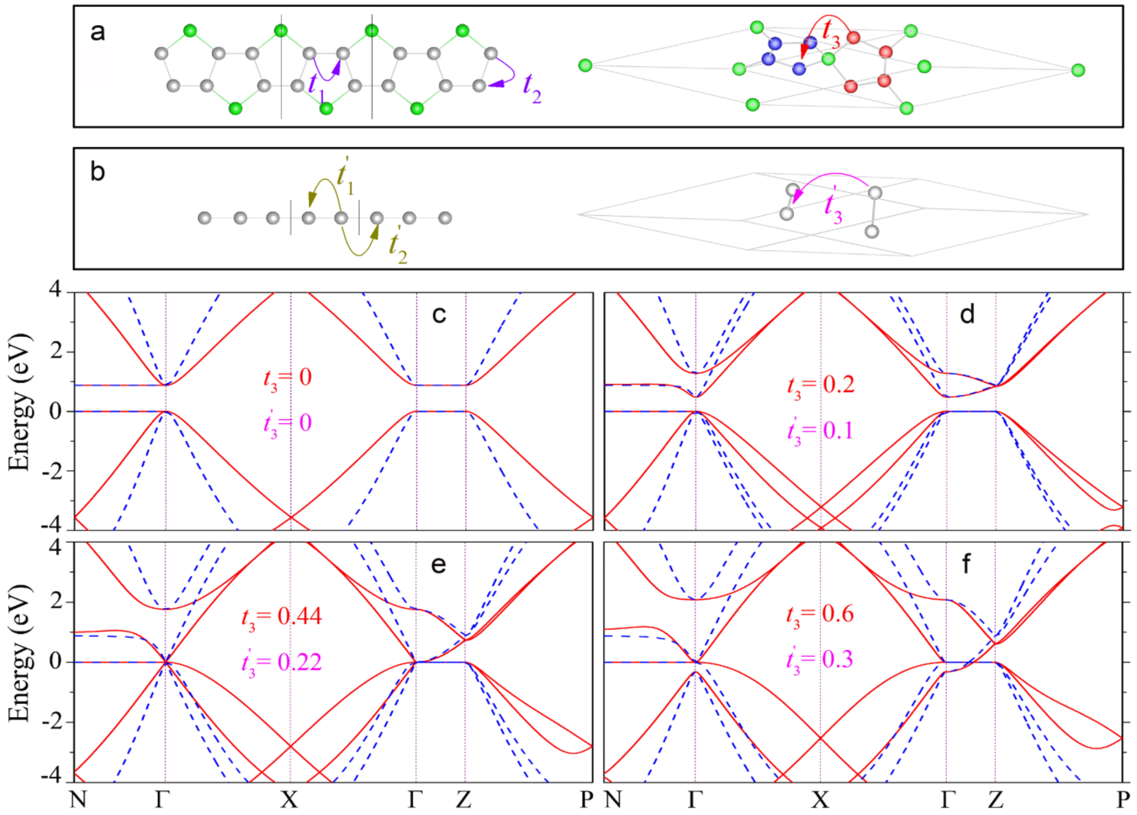

Supplementary Figure 4 | Comparison between 8-site TB model and 4-site TB model. (a) The original 8-site TB model and (b) the simplified 4-site TB model. (c-f) The band structures of the two TB models with different t_3 (t_3') values. The red solid lines and the blue dashed lines stand for the results of the original and the simplified TB models, respectively. In all case, t_1 (t_1') and t_2 (t_2') are set to 3.04 eV and -2.6 eV, respectively.

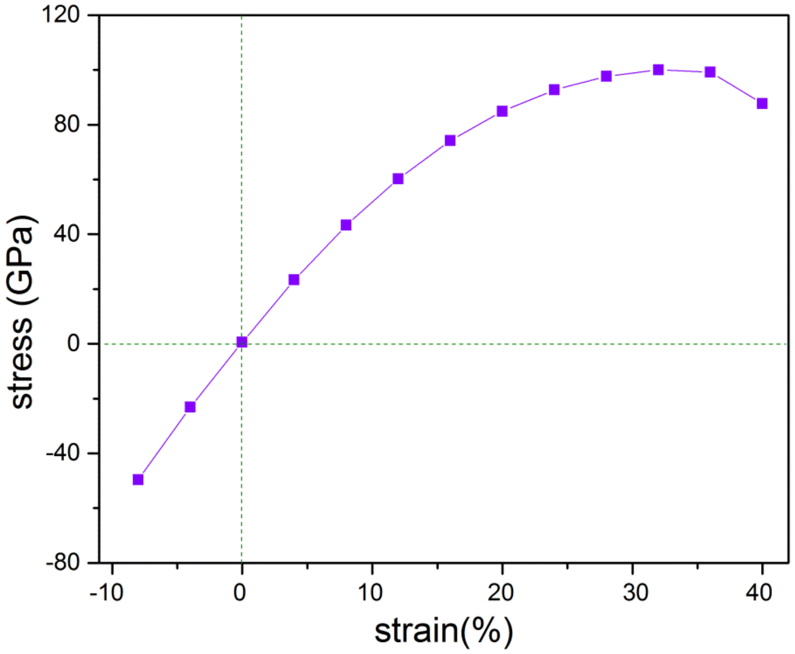

Supplementary Figure 5 | Strain-stress curve. Strain-stress relation curve of Pentagon Carbon for uniaxial strain along *z*-axis, showing a linear elastic regime up to 10% strain.

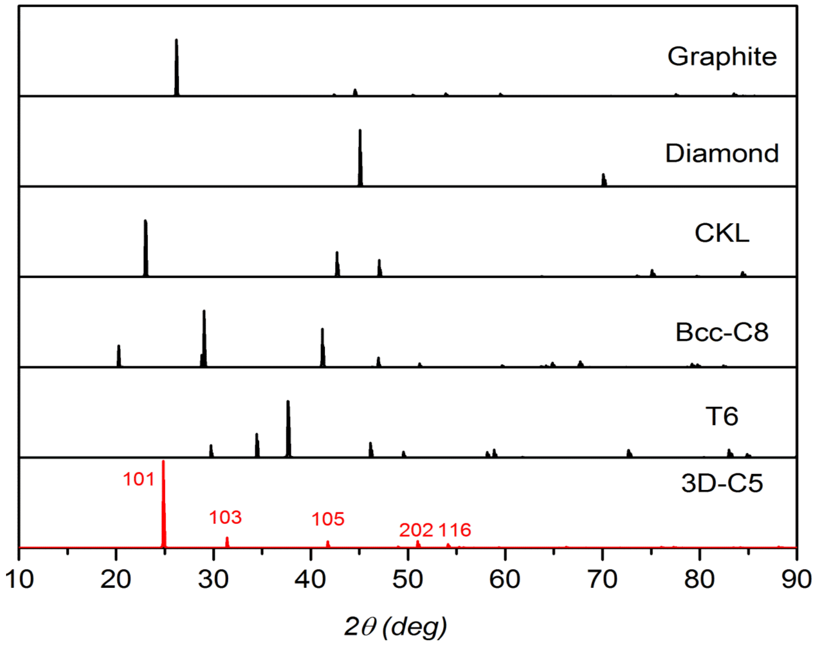

Supplementary Figure 6 | Simulated XRD patterns for representative carbon allotropes. To guide the experimental identification of Pentagon Carbon, we simulated its X-ray diffraction (XRD) pattern along with graphite, diamond, CKL, Bcc C8, T6, and 3D-C5. The simulated XRD of Pentagon Carbon (3D-C5) has a very strong peak around 24.86° correspond to the (101) diffraction. The other peaks (103), (105), (202), and (116) correspond to 2θ = 31.48, 41.75, 51.01, and 54.11, respectively.

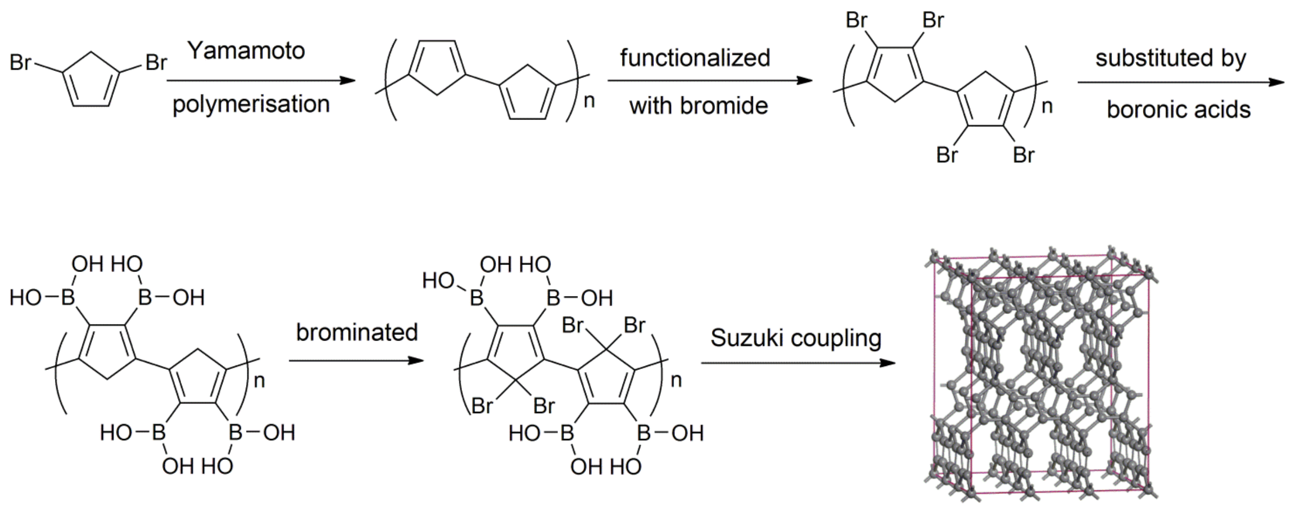

Supplementary Figure 7 | A possible reaction route for the chemical synthesis of Pentagon Carbon. First, a 1D ribbon composed of cyclopentadiene building units is synthesized by the Yamamoto polymerisation[10, 11] of a 1,4-dibromocyclopentadiene. The 1D ribbon is functionalized with bromide by Friedel–Crafts reaction[12], which is then substituted by boronic acids. The resulting product is further brominated and connected into the predicted 3D framework through Suzuki coupling[13, 14]. Each step in this process is either already achieved or have a high chance because of the existence of similar reactions.

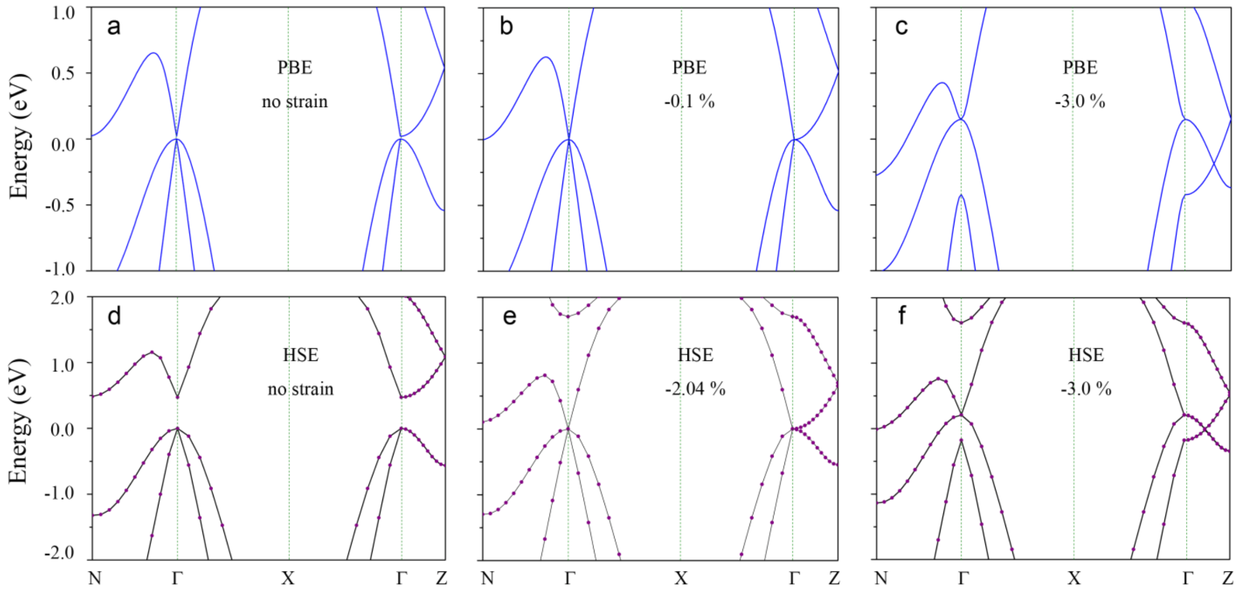

Supplementary Figure 8 | Band structures calculated with PBE and HSE under different strains. (a-c) Band structure of Pentagon Carbon under uniaxial strain of (a) 0%, (b) -0.1 %, and (c) -3% at PBE level. (d-f) Result using the hybrid functional (HSE06) with uniaxial strain of (d) 0%, (e) -2.04 %, and (f) -3%. The quantum phase transition is observed at the critical strain of -2.04 % on the HSE06 level.

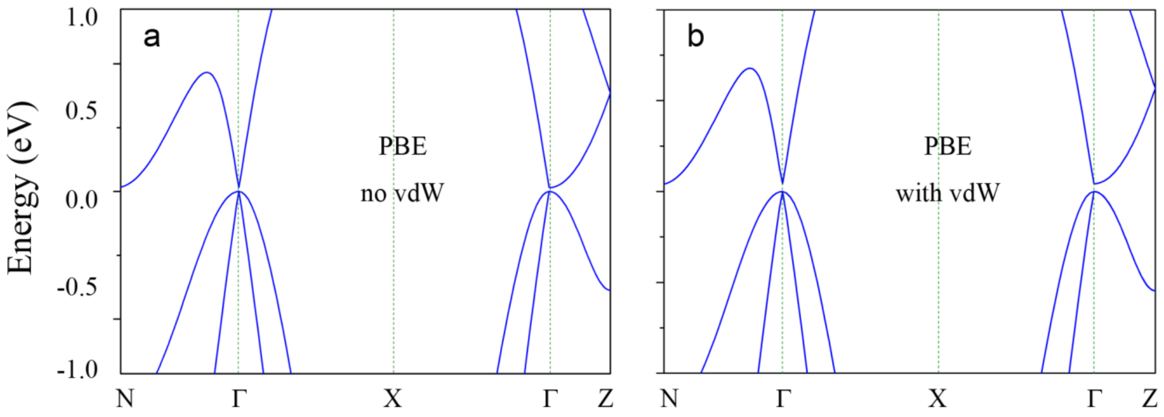

Supplementary Figure 9 | Comparison between calculations with and without van der Waals correction. The band structure of Pentagon Carbon (a) without and (b) with van der Waals forces. The effect of van der Waals (vdW) interaction is included in the DFT calculation with the method proposed by Grimme[9]. Since Pentagon Carbon is a 3D structure with covalently bonding, vdW interaction expected to have minor effects in the calculation results. For example, by the inclusion of vdW interaction, the cohesive energy is decreased from -8.79 eV to -8.89 eV (or by 1.1%) and the lattice parameters are decreased by about 0.2%. The main features of the band structure of Pentagon Carbon are not changed, only the bandgap is increased to about 40.5 meV with the inclusion of vdW interaction.

Supplementary Note 1: Lattice structure and stability

There are two irreducible atomic Wyckoff positions: one is the 16*h* (-0.3156, -1.0, -0.0738) site occupied by the sp^2 hybridized carbon atoms; and the other is the 4*a* (0.0, -1.0, 0.0) site occupied by the sp^3 hybridized carbon atoms. The comparison of cohesive energies as a function of volume for several representative carbon allotropes is shown in Supplementary Fig. 1a. One observes that the cohesive energy of Pentagon Carbon (3D-C5), although slightly higher than the most stable graphite and diamond, is less than T6[1] and Bcc-C8[2, 3] and is comparable with the CKL[4, 5] structure. For its mechanical property, we have calculated the elastic constant tensor in the linear elastic regime. For a tetragonal lattice system, only elastic constants C_{11}, C_{12}, C_{13}, C_{33}, C_{44} and C_{66} are independent, which are obtained as (unit GPa): C_{11} = 459.66, C_{12} = 112.8, C_{13} = 128.3, C_{33} = 672.39, C_{44} = 113.15, C_{66} = 9.42. We confirm that all these independent elastic constants satisfy the Born stability criteria[6, 7]. To further examine the dynamical stability of Pentagon Carbon, we perform calculations of its phonon spectrum. As shown in Supplementary Fig. 1b, there is no soft mode in the phonon spectrum, indicating that the proposed Pentagon Carbon structure is also dynamically stable.

Supplementary Note 2: Tight-binding model analysis

To capture the origin of the low-energy states, in Supplementary Fig. 2, we plot the projected density of states (PDOS) corresponding to the orbitals on the C1 and C2 atoms (c.f. Fig. 1c). One can find that the states around the Fermi level are mainly distributed on the C1 atoms, especially on their p_y/p_z orbitals (i.e. π orbitals), and the contribution from C2 atoms is almost negligible.

Based on the orbital analysis, we may construct a tight-binding (TB) model based on a single orbital per C1 site to capture the band features,

$$H = \sum_{<i,j>} \sum_{\mu} t_{ij} e^{-i\mathbf{k}\cdot\mathbf{d}_{ij}^{\mu}} \quad \text{Supplementary Equation 1}$$

where $i, j \in \{1,2,3\ldots 8\}$ label the eight C1 sites in a unit cell, $\mathbf{d}_{ij}^{\mu}$ is the displacement vector directed from site j to site i, t_{ij} is the hopping energy between sites i and j, and μ runs over all equivalent lattice sites under translation. We include three hopping processes: two are the nearest-neighbor hoppings along the armchair chains (t_1 and t_2); the third is between the two nearest sites of neighboring armchair chains (t_3), as indicated in Supplementary Fig. 3a. This simple model can indeed well capture the main features of the DFT result. It needs to be pointed out that the inter-chain coupling t_3 plays an important role and cannot be neglected: without t_3, the band structure is that of uncoupled interpenetrated armchair chains and is known to be a semiconductor with a sizable gap (see Supplementary Fig. 3b); finite t_3 is required for decreasing the gap and to reproduce the observed band-splitting in the DFT result (e.g. along Γ-X, see Supplementary Fig. 3c-3e). In order to fit the DFT band structure better, one can include two more higher-order hopping processes, t_4 and t_5, as indicted in Supplementary Fig. 3a. As one can see from Supplementary Fig. 3c, the inclusion of t_4 and t_5 makes the fitting of band dispersion along N-Γ and Γ-Z more accurate. The hopping parameters of Fig. 2a in the main text are $t_1 = 2.78$ eV, $t_2 = -2.43$ eV, $t_3 = -0.34$ eV, $t_4 = 0.15$ eV and $t_5 = 0.07$ eV, respectively. The values of t_4 and t_5 are reasonable for carbon-based materials. In

[PRB.78.205425 (2008)][8], the authors gave the third-nearest-neighbor tight-binding parameters for graphite and few-layer graphene. The distances of γ_1 and γ_2 are 3.35Å and 6.7 Å, and correspondingly, the values of tight-binding parameter for γ_1 and γ_2 are 0.35 eV and -0.0105 eV, respectively. In our TB model, the distances of t_4 and t_5 are 3.715 Å and 5.601 Å, and their magnitudes of hopping energy are 0.16 eV and 0.04 eV, respectively.

We also mention that the tight-binding model may be further simplified by modeling each four-site armchair chain with a dimer chain, and the obtained simplified model can also give a reasonably good result compared with the DFT band structure. As we have mentioned in the main text, the 3D Pentagon Carbon can be viewed as consisted of orthogonal pentagon-rings ribbons, the unit cell of pentagon-ring ribbon has four C1 atoms and two different nearest-neighbor hopping energies along the armchair chain, t_1 and t_2 as depicted in the left subfigure of Supplementary Fig. 4a. In effect, we can simplify this armchair chain into a dimer atom chain with two effective hopping energies $t_1^{'}$ and $t_2^{'}$ as shown in the left subfigure of Supplementary Fig. 4b. As for 3D Pentagon Carbon, the eight atoms TB model can be simplified to an effective TB model with four sites, at the same time, the t_3 transform to an effective hopping energy $t_3^{'}$ as denoted in right subfigure of Supplementary Fig. 4b. Eventually, the eight-site TB model can be simplified to a four-site TB model. As can be seen from Supplementary Fig. 4c-4f, these two TB models produce similar results, and both can catch the main features of the DFT band structure.

Supplementary Note 3: Results of hybrid functional method

The band structure results are verified by using the hybrid functional (HSE06) method. The comparison between PBE and HSE06 results are shown in Supplementary Fig. 6. One observes that the key features of the band structure and the phase transition are unchanged, as these are in fact dictated by the symmetry of the system. The main difference is in the quantitative band dispersion, the bandgap value, and the value of critical strain. As shown in Supplementary Fig. 6, for the unstrained system, the HSE06 bandgap is about PBE bandgap is 471.3 meV, larger than the PBE bandgap (~21.4 meV). The critical strain is also changed to about -2.04 % from the value of -0.1 % on the PBE level.

Supplementary References:

1. Zhang S., Wang Q., Chen X., Jena P. Stable three-dimensional metallic carbon with interlocking hexagons. *Proc. Natl. Acad. Sci.* **110**, 18809-18813 (2013).

2. Johnston R. L., Hoffmann R. Superdense carbon, C8: supercubane or analog of .gamma.-silicon? *J. Am. Chem. Soc.* **111**, 810-819 (1989).

3. Liu P., Cui H., Yang G. W. Synthesis of Body-Centered Cubic Carbon Nanocrystals. *Cryst. Growth Des.* **8**, 581-586 (2008).

4. Chen Y., *et al.* Carbon kagome lattice and orbital-frustration-induced metal-insulator transition for optoelectronics. *Phys. Rev. Lett.* **113**, 085501 (2014).

5. Zhong C., Xie Y., Chen Y., Zhang S. Coexistence of flat bands and Dirac bands in a carbon-Kagome-lattice family. *Carbon* **99**, 65-70 (2016).

6. Nye J. F. *Physical properties of crystals*. Clarendon Press (1985).

7. Wu Z.-j., Zhao E.-j., Xiang H.-p., Hao X.-f., Liu X.-j., Meng J. Crystal structures and elastic properties of superhard IrN2 and IrN3 from first principles. *Phys. Rev. B* **76**, 054115 (2007).

8. Grüneis A., *et al.* Tight-binding description of the quasiparticle dispersion of graphite and few-layer graphene. *Phys. Rev. B* **78**, 205425 (2008).

9. Grimme S. Semiempirical GGA‐type density functional constructed with a long‐range dispersion correction. *J. Comput. Chem.* **27**, 1787-1799 (2006).

10. Tsujii Y., Ohno K., Yamamoto S., Goto A., Fukuda T. Structure and Properties of High-Density Polymer Brushes Prepared by Surface-Initiated Living Radical Polymerization. In: *Surface-Initiated Polymerization I* (ed^(eds Jordan R). Springer Berlin Heidelberg (2006).

11. Yamamoto Y., Irie M., Hayashi K. Photoinduzierte Polymerisation. 6. Mitt.

Molekulargewichtsverteilung bei der kationischen Polymerisation von α-Methylstyrol. *Colloid Polym. Sci.* **256**, 396-396 (1978).

12. Olah G. A., Reddy V. P., Prakash G. Friedel‐Crafts Reactions. *Kirk-Othmer Encyclopedia of Chemical Technology*, (1980).

13. Old D. W., Wolfe J. P., Buchwald S. L. A Highly Active Catalyst for Palladium-Catalyzed Cross-Coupling Reactions: Room-Temperature Suzuki Couplings and Amination of Unactivated Aryl Chlorides. *J. Am. Chem. Soc.* **120**, 9722-9723 (1998).

14. Kim S.-W., Kim M., Lee W. Y., Hyeon T. Fabrication of Hollow Palladium Spheres and Their Successful Application to the Recyclable Heterogeneous Catalyst for Suzuki Coupling Reactions. *J. Am. Chem. Soc.* **124**, 7642-7643 (2002).
